# Microscopy and molecular biology for the diagnosis and evaluation of malaria in a hospital in a rural area of Ethiopia

**DOI:** 10.1186/1475-2875-11-199

**Published:** 2012-06-13

**Authors:** Maria A Santana-Morales, Raquel N Afonso-Lehmann, Maria A Quispe, Francisco Reyes, Pedro Berzosa, Agustin Benito, Basilio Valladares, Enrique Martinez-Carretero

**Affiliations:** 1 University Institute of Tropical Diseases and Public Health of the Canary Islands University of La Laguna, Tenerife, Spain; 2 National Centre of Tropical Medicine, Institute of Health Carlos III, Madrid, Spain; 3 Gambo General Rural Hospital, Shashemane, Ethiopia

**Keywords:** Diagnosis, Prevalence, Malaria, Ethiopia

## Abstract

**Background:**

Malaria is a leading public health problem in Ethiopia. Accurate diagnosis of *Plasmodium* infections is crucial for the reduction of malaria in tropical areas and for epidemiological studies. The role of light microscopy (LM) as gold standard has been questioned and, therefore, new molecular methods have been developed for the detection of *Plasmodium* species. The aim of the present work was to compare different malaria diagnostic methods in order to detect the most common species of *Plasmodium* and to broaden the knowledge of malaria prevalence in a hospital in a rural area in Ethiopia.

**Methods:**

A cross-sectional survey of 471 individuals was carried out in a hospital in the rural area of Gambo (Ethiopia). Blood samples were prepared for microscopic observation and collected in filter paper for Seminested-Multiplex PCR (SnM-PCR) and real time PCR (qPCR) testing. The SnM-PCR was considered as the gold standard technique and compared with the rest. Thus, agreement between SnM-PCR and LM was determined by calculating Kappa Statistics and correlation between LM and qPCR quantification was calculated by pair-wise correlation co-efficient.

**Results:**

Samples analysed by LM and SnM-PCR were positive for *Plasmodium* sp. 5.5% and 10.5%, respectively. Sensitivity was 52.2% by LM and 70% by qPCR. Correlation co-efficient between microscopy counts and qPCR densities for *Plasmodium vivax* was R^2^ = 0.586. Prevalence was estimated at 7% (95% CI: 4.7–9.3). *Plasmodium vivax* was the dominant species detected and the difference was statistically significant (*χ*^2^ = 5.121 *p* < 0.05). The highest prevalence of the parasite (10.9%) was observed in age groups under 15 years old.

**Conclusion:**

Accurate malaria diagnostic methods have a great effect in the reduction of the number of malaria-infected individuals. SnM-PCR detection of malaria parasites may be a very useful complement to microscopic examination in order to obtain the real prevalence of each *Plasmodium* species. Although SnM-PCR shows that it is a good tool for the determination of *Plasmodium* species, today light microscopy remains the only viabletool for malaria diagnosis in developing countries. Therefore, re-inforcement in the training of microscopists is essential for making the correct diagnosis of malaria. *Plasmodium vivax* was the predominant species in Gambo, a meso-endemic area for this species.

## Background

Malaria is the most prevalent tropical infectious disease in the world. An estimated 300–500 million people are annually infected with malaria, resulting in 1.5–3 million deaths [1]. Malaria mostly affects children in highly endemic areas with stable transmission. In areas with low or moderate endemicity, all age groups are affected and such areas are at a special risk of severe epidemics [2,3].

In Ethiopia, malaria is a leading public health problem where an estimated 68% of the population lives in malarious areas and three-quarters of the total land mass is regarded as malarious [4]. *Plasmodium falciparum* and *Plasmodium vivax* are the two predominant malaria species, accounting for 60% and 40% of malaria cases, respectively [5]. Malaria transmission follows a seasonal pattern (September-November), depending on the altitude and rainy season. Moreover, epidemic malaria is common, particularly in the highlands (1,000–2,000 m above sea level). However, many areas in the south and west of the country have a rainfall season beginning earlier in March and May or have no clearly defined rainfall season [5].

Therefore, accurate diagnosis of *Plasmodium* infections is crucial to reduce morbidity and mortality in tropical areas [6]. Studies of epidemiology and immunity depend on accurate detection, diagnosis, and density estimation.

Traditionally, light microscopy (LM) examination of blood smears has been considered the gold standard for the diagnosis of malaria [7]. LM has clear advantages since it incurs low costs, allows species identification and quantification and neither complex sample preparation nor advanced technology is required [8]. However, the role of LM as gold standard has been questioned due to false negative results at low levels of parasitaemia, with a predicted limit of detection of five to 20 parasites per microlitre of blood and frequent errors in species identification in mixed infections [9,10]. In this sense, microscopy seems to be an imperfect reference standard. Therefore, it is difficult to estimate its true sensitivity and specificity or to evaluate new diagnostic methods [11]. Several molecular methods based on the amplification of DNA have been developed for the detection of malarial infections in humans [12-16]. The semi-nested multiplex malaria PCR (SnM-PCR) is a widely used method and it is considered a molecular gold standard due to its good performance in the detection of mixed species infection and the ability to differentiate the four species of *Plasmodium*[15,16]. In recent years, new molecular methods have been developed for the detection of *Plasmodium* species, mostly based on real time quantitative PCRs (qPCR) [17,18]. These new molecular methods have been promoted as an automated, quantitative, and closed system that reduces the risk of cross-contamination inherent in conventional PCR [19]. Several real-time PCR methods for malaria have been described and validated within a research setting with high sensitivity and specificity values [20]. However, some advantages should be considered such as their ability to detect mixed *Plasmodium* infections but also some limitations, such as their application in rural areas without adequate laboratory conditions [19].

Alternative methods sensitive enough to detect low levels of parasitaemia in asymptomatic infections are required to complement or replace parasitological examination with light microscopy [21]. This would allow minimization of errors in diagnosis (false positives, false negatives and species misidentification) that may lead to biased estimates of protective efficacy against the parasite. The incorporation of molecular tools for the characterization of parasite infections has allowed an increase sensitivity in the detection of human malarial parasites in blood [22].

The aim of the present study was to compare different malaria diagnostic methods and to broaden the knowledge of malaria prevalence in a hospital in Gambo, Ethiopia.

## Methods

### Study site and cross-sectional survey

The study took place at Gambo General Rural Hospital. The hospital is located in the province of West Arsi, Ethiopia, which lies at 7°18′20.82″ north latitude and 38°48′55.37″ east longitude. The study was carried out from January to May 2010 and was approved by the Ethical Committee of Gambo General Rural Hospital.

A sample of 306 adults aged ≥16 years and 165 children aged ≤15 years was randomly selected from Gambo General Rural Hospital patients. The samples were divided in 192 women whose mean age was 32 years (range 16 to 89 years), 114 men, whose mean age was 40 years (range 16 to 98 years) and 165 children, whose mean age was five years (range one to 15 years). Pregnant women were excluded due to hospital policy. Microscopy was performed at the laboratory of the above-mentioned hospital and PCRs were performed at the University Institute of Tropical Diseases and Public Health of the Canary Islands.

### Laboratory methods

#### Blood sample collection and light microscopy observation

Finger-prick blood samples were collected and both thick and thin blood smears were prepared for microscopic observation. During the study period temperature values were around 25°C; samples were dried at room temperature before storage and were shopped at 4°C for further analysis. Thin and thick films were stained with a 10% Giemsa solution. A minimum of 200 microscopic fields were examined at a magnification of 1,000X using oil immersion optics before a slide was declared negative for malaria parasites by LM. Parasitaemia per microlitre of blood was estimated from the thick films as follows: the number of parasites per 200 white blood cells was multiplied by 8,000 (an average white blood cell count per microlitre) and then divided by 200. Routinely slides were read twice. Discordant results were evaluated by a third slide reading. Final species diagnosis was based on the majority agreement between experienced microscopists at Gambo General Rural Hospital.

Finger-prick samples on 3MM filter paper were obtained for the PCR assays. Each filter paper specimen was stored in a plastic bag at room temperature and shipped to the Canary Islands.

### DNA template extraction and amplification

DNA extraction was performed using commercial kits (Speedtools tissue DNA Extraction Kit, Biotools,Madrid, Spain). The seminested PCR was considered as the molecular gold standard. Therefore, suspected *Plasmodium* species by LM were confirmed by semi-nested-multiplex PCR [22] and by qPCR using primers and probes developed by commercial kits (PrimerDesign^TM^Ltd, Alicante, Spain), which amplify the plasmepsin 4 gene in *P. falciparum* and the aspartic protease PM4 gene in *P.vivax*. Each reaction contained 5 μL DNA, 10μL 2xPrecision^TM^MasterMix (Primerdesign), 1μL Pathogen Primer/Probe mix, 1μL Internal extraction control primer/probe mix and 3 μL RNAse/DNAse free water. The thermal profile used was 15 minutes at 37°C, followed by 10 minutes at 95°C and 50 cycles of 10 seconds at 95°C and 1 minute at 60°C. A second qPCR was performed as previously described by Rosanas-Urgell *et al.*[6]. All PCRs were performed two times for each sample.

### qPCR validation

Evaluation of qPCR efficiency and reproducibility was performed on standard curves using two positive control plasmids with the respective inserted amplicons available with the commercial kit. Geometric mean and standard deviation were calculated from triplicates in three independent assays. The standard curve for each *Plasmodium* species was made from a 10-fold serial dilution of the control plasmids ranging from 20^5^ copies/μL to 2 copies/μL. Amplification efficiencies for the different primer pairs and probes were calculated with the formula: Efficiency = 10(-1/Slope) -1.

The amount of target in an unknown sample was quantified by converting the threshold cycle (Ct) into template copy number by using the two standard curves. Samples yielding Ct values equal or higher than 40, were considered negative for *Plasmodium* species [23]. All protocols were performed following manufacturers instructions two times.

### Statistical analysis

Results from SnM-PCR were compared with those from LM and qPCR. In this study, SnM-PCR was used as a gold standard. Agreement between SnM-PCR and LM was determined by calculating Kappa Statistics with 95% confidence intervals. Values were interpreted with the Landis and Koch classification [24] as follows: k = 0.41–0.60, moderate agreement, k = 0.61–0.80, good agreement; k = 0.81–1.00, almost perfect agreement beyond chance. Correlation between LM and qPCR quantification was calculated by pair-wise correlation co-efficient.

Prevalence was calculated by dividing the number of positive samples by the total number of tested samples. The age trends of the different categorical variables were assessed using the Chi-square test for linear trend.

All statistical calculations were performed with SpSS19 statistical software and *P* values <0.05 were considered statistically significant.

## Results

The study included 417 samples from Gambo General Rural Hospital. Samples analysed by LM and SnM-PCR were positive for *Plasmodium* sp. 5.5% and 10.5%, respectively. None of the parasite positive samples by microscopy was negative by SnM-PCR while 5% of the negative samples by LM were positive by SnM-PCR. Overall LM sensitivity was only 52.2%, being 58.8% for *P. vivax* and 33.3% for *P. falciparum* (Table 1). In children, the sensitivity was 66.6% for *P. vivax* and 20% for *P. falciparum*. The concordance between both microscopy and SnM-PCR by the Kappa co-efficient showed a good agreement (k = 0.62).

**Table 1:** Comparative study of the sensitivity of the LM and qPCR using SnM-PCR as gold standard

	**Sensitivity**		
	**Total**	***P. vivax***	***P.falciparum***
**LM**	52.2%	58.8%	33.3%
**qPCR**	70%	82.3%	33.3%

All SnM-PCR-positive samples were analysed by qPCR. Overall, sensitivity was 70%, being 82.8% for *P. vivax*and 33.3% for *P. falciparum* (Table 1).

**Figure 1:**
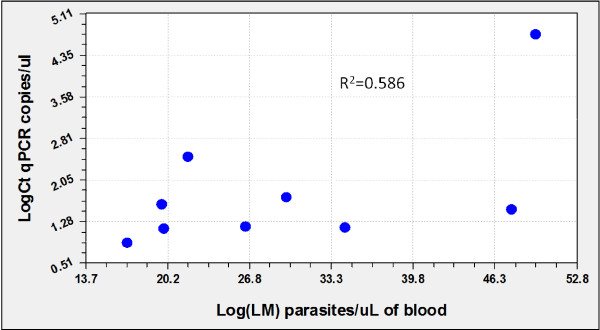
** Comparison of quantification assessed by LM *versus *qPCR(PrimerDesign**^**TM**^**Ltd).**

The geometric mean parasite density was 2,274 parasites/μL (range 680 to 8,000 parasites/μL) by microscopy. By qPCR (PrimerDesign^TM^Ltd) the geometric mean parasite density was 5.6 copies/μl of blood (ranging from 0.78 to 279.23 copies/μl of blood). Correlation co-efficient between microscopy counts and qPCR densities for *P. vivax* was R^2^ = 0.586 (Figure 1). Positive samples were analysed by the qPCR designed by Rosanas-Urgell *et al.*[6]. The correlation co-efficient between both qPCRs in *P. vivax* was R^2^ = 0,980. These correlation co-efficients could not be estimated for *P. falciparum* since only ten samples were positive by light microscopy. 

A total of 471 samples, selected as defined above, were included in the cross-sectional malaria study. Based on PCR results, weighted parasite prevalence was estimated at 7% (95% CI: 4.7–9.3). The prevalence of *P. falciparum* was 2.1% (95% CI: 0.81–3.4) while that of *P. vivax* was 5% (95% CI: 2.9–6.7). *Plasmodium vivax* was the dominant species detected and the difference was statistically significant (*χ*^2^ = 5.121 *p* < 0.05). Malaria prevalence in children was lower than in adults and the difference was statistically significant (χ ^2^ = 5.93 *p* < 0.05) (Table 2).

**Table 2:** Malaria prevalence by sex and age

***Plasmodium* species identified**	**Children**	**Female**	**Male**	**χ **^**2**^	***P*-value**
*P. falciparum*N(%)	6(3.6)	4(2.1)	0	−	−
*P. vivax*N(%)	12(7.3)	8(4.2)	3(2.6)	−	−
Total N(%)	18(10.9)	12(6.25)	3(2.6)	5.93	0.01

**Figure 2:**
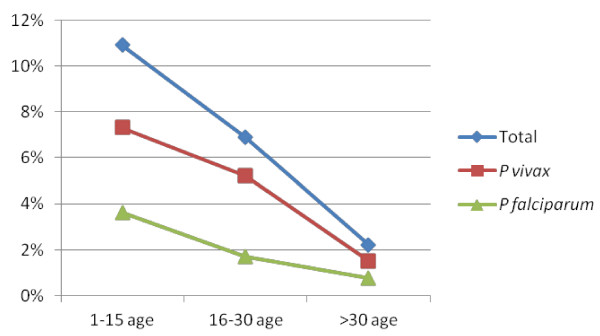
** Prevalence pattern of *Plasmodium *species among different age groups.**

Among tested adult individuals, infection frequently occurred before 30 years of age and only in three adults were parasitized at an age over 30 years old. The highest prevalence of the parasite (10.9%) was observed in age groups under 15 years old with a mean age of five years (Figure 2).

## Discussion

Ethiopia includes regions of differing malaria endemicity and transmission. The peak of malaria incidence follows the main rainfall season (July-September) each year. However, many areas in the south and west of the country have a rainfall season beginning earlier in March and May or have no clearly defined rainfall season [25]. Futhermore, the sample collection was carried out during the dry season in this area.

A SnM-PCR was considered and used in this study as a molecular gold standard. The prevalence obtained by SnM-PCR was 7%, higher than the estimated by LM method, which demonstrates an increased sensitivity with respect to the latter. In this regard, it is noteworthy that all microscopy-positive samples were also found to be positive by SnM-PCR. However, the SnM-PCR test was able to detect positive samples that were missed by microscopy.

In this study a high number of children (80%), who initially had been negative for LM method, were later found to be infected with *P. falciparum* by SnM-PCR. These undetected sub-microscopic infections do not develop the disease in the patients but they may have an enormous impact on malaria transmission in endemic areas. Infected individuals may play the role of parasite reservoir since their blood is able to infect mosquito vectors and they may reintroduce malaria into certain regions [26,27]. Molecular methods have potential use to detect malaria parasites in asymptomatic infections, mainly in children infected with *P. falciparum*, which causes the most severe malaria infection.

The qPCR sensitivity was 70% when compared to SnM-PCR. Despite measurements taken over 45 cycles in qPCR, previous studies and the manufacturers consensus rule was considered to decide whether a sample was positive or not (Ct value <40 [23]).

The qPCR is the most analytically sensitive method (sensitivity 70%), followed by microscopy (sensitivity 52.5%), when compared to SnM-PCR. Overall, qPCR shows substantial agreement with other molecular techniques for the detection of *Plasmodium* prevalence. The SnM-PCR has been reported for the diagnosis of low-level parasitaemia, and real-time PCR for the diagnosis of high-level parasitaemia. In countries where malaria is not endemic and where it has recently re-emerged, there is lack of physicians and microscopy-skilled laboratory staff specializing in malaria, which makes the diagnosis of malaria difficult. Therefore, SnM-PCR is a good alternative since it does not require special training for interpretation of the results, the opposite to microscopy, in which specific training is needed for species differentiation. In addition, SnM-PCR is not altered by the subjectivity of the observer [22].

The advantage of qPCR to other molecular techniques is the quantification of parasite densities. The correlation between quantifications by both qPCR showed a high correlation (R^2^ = 0.980) confirming that qPCR using commercial kits (PrimerDesign^TM^Ltd) is an accurate method to quantify parasite densities. When correlating quantification by qPCR with LM counts in samples where both techniques showed positive results, a low correlation for *P. vivax* (R^2^ = 0.586) was found. This low value might be due to overall lower densities, possibly around the detection limit in *P. vivax* infections, because the scarcity of the template in case of a very low parasite density is expected to lead to imperfect detection [6]. Another possible explanation would be the presence of schizonts in the blood, because clinical samples with similar levels of parasitaemia by LM but with different proportions of schizonts (14–21 genomes) will vary in copy number and consequently in real-time PCR parasite quantification. In this sense, the comparison of parasitaemia determined by LM with that assessed by genomic standard DNA quantification curve showed significantly divergent results among malaria samples [28].

Microscopic diagnosis is considered to be the reference standard for determining the protective efficacy of prophylactic drugs or vaccines. However, microscopy is an imperfect reference standard with many inherent limitations [29]. The difficulties in correctly identifying low-density infection may contribute to the discrepancy in microscopy results. Furthermore, these discrepancies indicate a need to implement a rigorous quality assurance system within the routine laboratory diagnostic techniques for malaria in Ethiopia. Molecular techniques, such as PCR, have a lower detection threshold for *Plasmodium* than microscopy [30,31], and it may be a more sensitive diagnostic tool in a population where low-density infections are expected. For this reason, the study addressing malaria situation in Gambo was carried out using SnM-PCR.

The prevalence observed was 7%, which is higher than the prevalence observed in previous surveys performed in Amhara (4.1%), Jimma (5.2%) and SNNP Regional States (5.4%) [32-34]. This is due to the fact that the prevalence value was obtained with SnM-PCR, which is a more sensitive technique than microscopy. The prevalence of *Plasmodium* species was 69.7% and 30.3% for *P. vivax* and *P. falciparum*, respectively, unlike the previous paradigm of *Plasmodium* species composition in Ethiopia (*P. falciparum* 60% and *P. vivax* 40% of total malaria cases) [5]. These differences seem to indicate that the proportion of both species is changing. A study, carried out from 2007 to May 2009. It is also important to highlight the fact that this study was carried out with patients admitted to a hospital in a rural area and thus this was a biased study. In the area of study a decrease in cases of *P. falciparum* mono-infection and an increase of *P. vivax* mono-infection have been previously reported [35] and another study, conducted in areas of Oromia in December 2009, found a greater proportion of *Plasmodium* infection due to *P. vivax*[36]. The result of the present study, carried out between January and May 2010, may be explained due to the tendency of *P. vivax* to cause long-term chronic infection [37] and to dominate during low transmission periods and dry season (March-June) [38].

In the present study, a significant association between children patients and number of positive blood smears (*χ*^2^ = 5.93 *p* < 0.05) was found. Where transmission is relatively stable and intense, the mean and median age of patients diagnosed with malaria at clinics is generally under five years old [39]. For this reason, due to such age-dependency of the infection, children acquire certain malaria immunity [40]. Studies reported that individuals living in areas of unstable and low intensity malaria transmission do not acquire significant immunity to the disease, and hence malaria infections can be observed in all age groups [41,42]. In the present study, the least affected age group was the group over 30 years old (9.1%). The epidemiological condition prevailing in Gambo from a prospective parasitological survey point of view suggests that the area is characteristic of a stable, moderate level of malaria endemicity being considered a meso-endemic area, based on the classification established by WHO [43].

## Conclusion

As might be expected, in the present study, both PCR detection protocols were more efficient methods than microscopic examination. SnM-PCR methodology seems to be the most sensitive method to detect *P. falciparum* and *P. vivax* from clinical blood spots dried on filter paper, avoiding losing 80% of infection by *P. falciparum*. This large difference (80% of positive individuals who were negative by light microscopy) suggests that microscopists are not always appropriately trained in the diagnosis of malaria. Therefore it is necessary to implement quality systems in laboratories to avoid inter-laboratory variability.

These results suggest that, in malaria endemic areas where transmission of both *P. falciparum* and *P. vivax* occurs, SnM-PCR detection of malaria parasites may be a very useful complement to microscopic examination in order to obtain the real prevalence of each species and accurate epidemiological data. The correct diagnosis of malaria parasite species can reduce the number of malaria-infected individuals who carry the parasites among populations.

The results of the present study also contribute to evaluate and broaden the knowledge of malaria in Gambo. Results of the cross-sectional study show 7% prevalence and a higher prevalence of *P. vivax* versus *P. falciparum*. In addition, this study shows a greater number of infected children compared to adults, suggesting that Gambo is a meso-endemic area.

## Competing interests

The authors declare that they have no competing interests.

## Authors’ contributions

MASM carried out the sample collection in Gambo (Ethiopia), the microscopy and molecular study, the analysis and interpretation of data and prepared the manuscript. RNAL and MAQ helped with the statistical analysis and interpretation. PB and AB helped with the performance of molecular studies. FR helped with the collection of samples. BV helped to draft the manuscript and EMC coordinated and funded the study and drafted the manuscript. All authors read and approved the final manuscript.
